# Economic Performance and Meat Quality Traits of Extensively Reared Beef Cattle in Greece

**DOI:** 10.3390/ani15111601

**Published:** 2025-05-29

**Authors:** Vasiliki Papanikolopoulou, Stella Dokou, Anestis Tsitsos, Stergios Priskas, Sotiria Vouraki, Angeliki Argyriadou, Georgios Arsenos

**Affiliations:** 1 Laboratory of Animal Production and Environmental Protection, School of Veterinary Medicine, Faculty of Health Sciences, Aristotle University of Thessaloniki, 54124 Thessaloniki, Greece; vipapani@vet.auth.gr (V.P.); stpriskas@vet.auth.gr (S.P.); svouraki@uoi.gr (S.V.); argyrian@vet.auth.gr (A.A.); 2 Laboratory of Animal Nutrition, School of Veterinary Medicine, Faculty of Health Sciences, Aristotle University of Thessaloniki, 54124 Thessaloniki, Greece; dokoustella@vet.auth.gr; 3 Laboratory of Animal Food Products Hygiene—Veterinary Public Health, School of Veterinary Medicine, Faculty of Health Sciences, Aristotle University of Thessaloniki, 54124 Thessaloniki, Greece; tsitanes@vet.auth.gr; 4 Laboratory of Animal Production, Nutrition and Biotechnology, Department of Agriculture, School of Agriculture, University of Ioannina, 47100 Arta, Greece

**Keywords:** extensive systems, grazing, beef quality, economic performance

## Abstract

Extensive cattle farming significantly contributes to Greece’s agricultural economy. In such systems, animals mainly graze on natural grasslands whose biodiversity significantly affects meat quality traits. In Greece, the sector faces several economic challenges, while the literature investigating beef quality produced by these systems is scarce. Hence, this study aimed to (i) evaluate farms’ economic performance; (ii) assess meat quality; and (iii) investigate the presence of heavy metals in liver samples of extensively reared beef cattle. The study involved three farms located in the Axios River Delta, a protected area of significant ecological importance in Northern Greece. A designated questionnaire was used to collect farm technical (herd size, meat production, grazing, feeding, reproduction, animal health) and economic data (income, variable costs). Meat samples of the *Longissimus dorsi* muscle (ninth rib) from 54 carcasses were collected and subjected to physicochemical (color, pH, texture, chemical composition, fatty acid profile) and microbiological analyses. Additionally, heavy metal analysis was conducted on 14 liver samples. A comparative analysis using parametric and non-parametric tests was performed to assess differences in meat quality traits between the 1st and 15th days of storage. The economic analysis showed that all studied farms operated with losses, with the average gross margin excluding subsidies being negative at EUR 130.5 ± 92.60/year per animal. Beef exhibited low fat content (1.1 ± 1.12%), with an average pH_24_ value of 5.5 ± 0.36, respectively. The concentrations of polyunsaturated, monounsaturated, and saturated fatty acids were 2.7 ± 0.72%, 44.6 ± 4.71%, and 47.3 ± 4.91%, respectively. Over the 15-day storage period, the yellowness (b*) value (*p* < 0.01), hue angle (*p* < 0.001), cohesiveness (*p* < 0.01), and springiness (*p* < 0.01) significantly decreased, while the lightness (L*) value significantly increased (*p* < 0.01). The mean Total Mesophilic Viable Counts and Total Enterobacterales were 5.0 log_10_ CFU/g and 2.34 log_10_ CFU/g, respectively, while heavy metal concentrations in bovine livers were below the maximum limits set by the European Commission. The results suggest that, despite the financial losses observed, beef’s improved color parameters during storage, along with other favorable quality traits, highlight the potential of extensive cattle farming to meet consumer demand and support value-added marketing.

## Simple Summary

Extensive cattle farming is important for Greece’s agricultural economy. However, the sector faces economic challenges and lacks research on the quality of beef produced. Hence, this study aimed to evaluate farms’ economic performance and assess meat quality and the presence of heavy metals in liver samples from cattle reared extensively in the Axios River Delta, a protected area of significant ecological importance in Northern Greece. Economic analysis was based on data collected by designated questionnaires, while meat samples were collected and subjected to physicochemical and microbiological analyses. The results showed that all farms were strongly dependent on subsidies to be economically viable. Furthermore, beef quality traits were improved over a 15-day storage period. Overall, this study suggests that despite farms’ low profitability, beef’s enhanced nutritional value and quality characteristics highlight the potential of extensive cattle farming to meet consumer demand and support value-added marketing strategies.

## 1. Introduction

Beef quality is crucial for consumer preferences and is influenced by a range of intrinsic and extrinsic factors. The former category includes animal-related factors, while extrinsic factors are associated with housing and management practices [1,2,3]. Among extrinsic factors, farming systems and feeding practices are critical in determining meat quality [4,5,6,7,8]. Globally, beef cattle are mainly reared under extensive or intensive farming systems. The differences between these systems lie in feeding and housing practices. Particularly, in extensive systems, animals have access to pasture, and feeding relies mainly on grazing. Supplementary feeding may be provided in cases where grass resources are insufficient. In contrast, beef under intensive systems are kept indoors and offered a high-energy grain-based diet [4].

Several studies have shown that meat produced from beef reared under extensive conditions exhibits superior meat quality traits, especially a healthier fatty acid (FA) profile [9,10,11,12,13]. Several authors confirmed that pasture-feeding diets contributed to beef production with higher concentrations of polyunsaturated fatty acids (PUFAs) and antioxidants (α-tocopherol, β-carotene), both of which improve overall meat quality [14,15]. Current consumer preferences for meat products are driven by healthier options with a lower environmental impact and in line with animal welfare. Several authors agree that beef produced by extensive systems satisfies these demands, including meat quality and ecological sustainability [9,11,16,17,18,19].

In Greece, extensive beef systems have significant social, economic, and environmental impacts. In such systems, cattle show great environmental adaptability, disease resistance, and low reliance on purchased feed, potentially contributing to higher profitability. Feeding is mainly based on exploiting natural pastures, whose biodiversity significantly contributes to meat quality. One of Greece’s most important ecosystems is the Axios River Delta, part of the Axios–Loudias–Aliakmonas Delta National Park in Central Macedonia, Northern Greece. Due to its ecological importance, the area is part of the European Natura 2000 protected network and is classified by the Union for Conservation of Nature (IUCN) as category VI, emphasizing the sustainable use of natural resources. The region remains largely natural and supports rich wildlife, with low population density practicing traditional activities such as livestock and mussel farming, as well as rice cultivation. Around 1000 cattle are raised extensively on three farms across grasslands, pastures, scrubs, and forests in a semi-arid climate [20]. However, while this area contributes positively to biodiversity and livestock farming, it also raises potential environmental and food safety concerns. Previous research has shown that river sediments, particularly in large river deltas, serve as important reservoirs of heavy metals from both natural and anthropogenic sources, contributing to the contamination of surrounding surface soils [21,22]. These contaminants can be absorbed by pasture plants and transferred to grazing livestock, potentially entering the human food chain and raising public health concerns.

Despite the significant benefits of extensive systems for meat quality and environmental sustainability, there is a lack of relevant literature in Greece. While some studies have explored the suckler cow sector and the economics of extensive beef farming in specific regions, data on meat quality from these systems remain scarce. [23,24]. To date, only one study has been conducted, which examined a limited number of meat samples produced by a specific breed, the Katerini breed, that is classified as at risk of extinction [25]. Furthermore, analyses of heavy metals in bovine biological samples have not been performed, despite the known role of river deltas as reservoirs of these metals. Notably, no research has explored the combined aspects of meat quality and safety and the economic viability of extensive systems. We hypothesized that meat produced by these systems, with unique nutritional traits from grazing, could support new market products reflecting the characteristics of extensive farming. This could contribute to the financial sustainability of Greece’s beef sector, motivating farmers to maintain their practices. Such a study also holds international relevance, offering valuable insights applicable to other similar extensive farming systems worldwide, particularly those operating within protected areas.

Thus, the present study aimed to assess the economic performance and meat quality of cattle reared under extensive systems in the natural grasslands of the Axios River Delta. The study also investigated the presence of heavy metals in liver samples as indicators of potential environmental exposure and food safety risk.

## 2. Materials and Methods

### 2.1. Study Area and Farms

**Figure 1:**
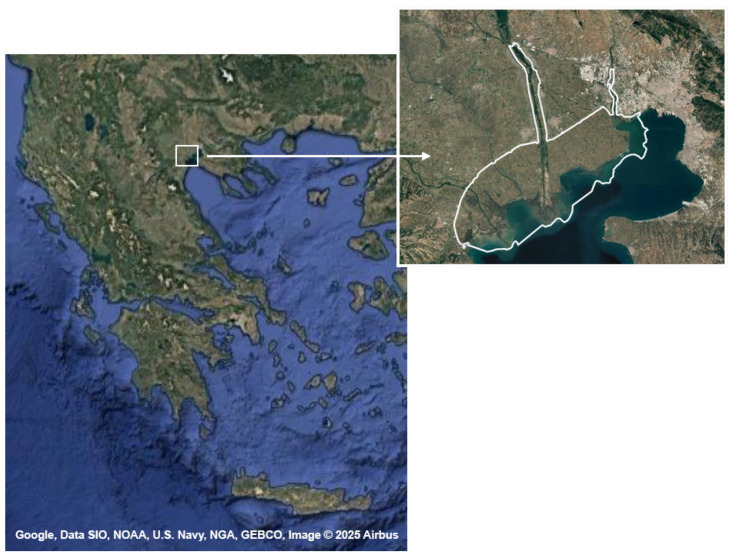
Map of Northern Greece illustrating the Axios–Loudias–Aliakmonas Delta National Park in which the study was conducted.

Three beef cattle farms located in the Axios River Delta, part of Axios–Loudias–Aliakmonas Delta National Park in Central Macedonia, Northern Greece (Figure 1), were included in the study. Farm A (FA) consisted of 500 cows and was classified as a large farm. In contrast, the other farms, Farms B (FB) and C (FC), were smaller, with 80 and 60 cows, respectively. FB and FC were operated primarily by family members, while in FA, the labor was based on employees. In all farms, animals were crosses of local breeds (Brachyceros, Katerini, Sykia) with the Limousin breed and reared under an extensive system. Calves were milk-fed until 5 months of age. Following weaning, their nutritional requirements were primarily met through grazing on natural grasslands, with supplementary feeding offered only during times of poor vegetation. The supplementary feed consisted of a concentrate composed of corn grain (20%), barley grain (40%), soybean meal (15%), and rice bran (25%). Additionally, straw was provided as a roughage source. The males were slaughtered at 18 months of age, with an average body weight (BW) of 550 kg.

### 2.2. Data Collection and Sampling

A group of veterinarians performed field visits using a designated questionnaire to collect technical and economic data on the studied farms (Supplement File S1_Dataset S1). Specifically, technical data included information regarding management practices such as herd size, meat production, grazing, feeding, reproduction, animal health, and welfare. Economic parameters regarding income (subsidies, meat production, and animal sales) and variable costs (feeding, labour, transportation, utility, land, veterinary expenses, and equipment) were also collected. Based on the above data, profitability (gross and net profit margin) and cost efficiency measures (variable costs) were calculated to evaluate farm economic performance.

Meat samples from 54 beef carcasses were collected and subjected to physicochemical and microbiological analyses. Specifically, the *Longissimus dorsi* muscle from the 9th rib was cut 24 h post-mortem from the cold carcasses. Additionally, 14 liver samples were collected for heavy metal analysis. The samples were transported to the laboratory in insulated polystyrene boxes maintained at ≤4 °C and stored in vacuum packaging under refrigeration (≤4 °C) for further analysis. The animals were slaughtered at the same commercial abattoir in Northern Greece between March 2023 and November 2024. The facility was licensed for the slaughter of ruminants and pigs and operated two distinct slaughter lines. The cattle slaughter line was entirely separate, while small ruminants and pigs were slaughtered in parallel. The abattoir had an hourly capacity of 10 cattle, 50 pigs, and 150 small ruminants, with an annual meat production of approximately 2500 tons.

### 2.3. Meat pH

Meat pH was measured on the 1st and 15th day of storage using a portable pH meter, the FiveGo pH Meter F2 (Mettler Toledo, Zaventem, Belgium). The measurement was conducted non-destructively by piercing a hole into each sample. For consistency, three consecutive readings were taken at the same incision point, and the average value was calculated. To ensure measurement accuracy, the instrument was calibrated prior to use with two standard pH buffer solutions, one at pH 4.00 and the other at pH 7.00.

### 2.4. Meat Color

Meat color was assessed on the 1st and 15th days of storage following the methodology described by Tsitsos et al. [26]. Color assessment was performed on freshly exposed surfaces of the meat samples immediately after unpacking. A CR-410 Chroma Meter (Konica Minolta, Tokyo, Japan) was used, with a 50 mm aperture size, illuminant C, and a 2° observer. Calibration was carried out with a white tile characterized by values of Y: 94.8, X: 0.3130, and y: 0.3190. Each sample was scanned three times at different positions perpendicular to the muscle fibers while avoiding areas with fat or connective tissue. The readings for lightness (L*), redness (a*), and yellowness (b*) were averaged for each sample. Based on these values, chroma and hue angle were calculated according to the formulas recommended by the American Meat Science Association (AMSA) [27].

### 2.5. Meat Tenderness

Texture profile analysis was performed on the 1st and 15th days of storage using a Stable Micro Systems TA.HDplus Texture Analyser (Stable Micro Systems, Godalming, UK) equipped with a flat-faced cylindrical probe of 1.27 cm diameter. The analyzer was connected to a computer running Exponent software (version 6.1.16.0). For the analysis, an oval-shaped piece with uniform dimensions of 2–3 cm in width and thickness was excised from the center of each sample. A double-compression cycle test was performed with the probe moving downward perpendicular to the muscle fibers. The test parameters included a pre-test speed of 1.00 mm/s and a test and post-test speed of 5.00 mm/s, achieving a 40% deformation of the sample height in each cycle. The time interval between two compression cycles was 2.02 s. The force–time plot generated by the software represented the resistance of the sample to compression over time, allowing for the calculation of key texture parameters, including hardness 1, hardness 2, cohesiveness, springiness, and chewiness, as described by Skaperda et al. [28].

### 2.6. Meat Chemical Composition and Fatty Acid Profile

Meat chemical analysis was conducted on the 15th day of storage, including the determination of its chemical composition (moisture, total fat, total protein, collagen, salt, and ash) and fatty acid profile.

Meat chemical composition was performed following the method described by Tsitsos et al. [29]. Briefly, 100 g of meat was weighed and placed on a plastic sample pan for analysis using a near-infrared spectrometer (NIR, Perten DA7250, Perkin Elmer Ltd., Waltham, MA, USA) calibrated for meat and meat products. The calibration process adhered to ISO and AOAC-approved methodologies, including Soxhlet extraction for fat, drying cabinet methods for moisture, Kjeldahl for protein, hydroxyproline for collagen, muffle furnace for ash, and ICP-MS for salt. Calibration models were developed using Artificial Neural Networks (ANNs) and Honigs Regression™ (PerkinElmer, Waltham, MA, USA) to ensure high accuracy and reliability of the measurements.

The meat fatty acid profile was determined using the Soxtherm Soxhlet Extraction System (C. Gerhardt GmbH & Co. KG, Konigswinter, Germany), in accordance with AOAC Method 991.36 [30], as described by Tsitsos et al. [29]. The extracted fatty acids were subjected to transesterification in a methanolic potassium hydroxide solution to produce fatty acid methyl esters (FAMEs). The FAMEs were analyzed using gas chromatography with flame ionization detection (GC-FID). Chromatographic analyses were performed on a Shimadzu GC-2010 Plus High-End gas chromatography system equipped with an FID detector (Shimadzu, Kyoto, Japan) and a Supelco SP2560 column (100 m × 0.25 mm × 0.20 μm) (Sigma-Aldrich, St. Louis, MO, USA). Helium gas with a purity of 99.999% was used as the carrier at a 2 mL/min flow rate. The injection volume was 1 μL with a split ratio of 1:50, and both the injector and detector temperatures were set at 250 °C. The oven temperature program began at 110 °C, held for 7 min, and increased gradually at 3 °C/min to 190 °C, where it was maintained for 2 min. The next phase involved an increase at 0.5 °C/min to 205 °C, followed by a 5 °C/min rise to 230 °C (held for 5 min), and a final step at 5 °C/min to 240 °C (maintained for an additional 5 min). The total run time for the chromatographic analysis was 82.67 min.

### 2.7. Heavy Metal Analysis

Heavy metal analysis in liver samples was conducted using an Inductively Coupled Plasma Mass Spectrometer (ICP-MS, Agilent 7850) (Agilent Technologies, Santa Clara, CA, USA) following the protocol outlined by the U.S. Food and Drug Administration [31]. Sample preparation involved acid digestion using high-purity nitric acid (HNO_3_) and hydrogen peroxide (H_2_O_2_). An aliquot of the sample was combined with the acids and subjected to microwave digestion at 210 °C for 60 min. After digestion, the samples were diluted appropriately and analyzed using the ICP-MS method to determine the concentrations of heavy metals.

### 2.8. Microbiological Analysis

The microbiological analysis of the meat samples was conducted on the 1st day of storage (24 h after slaughter). More specifically, Total Mesophilic Viable Counts (TMVCs) and the populations of Enterobacterales and *Escherichia coli* were assessed in accordance with EN ISO 4833-1:2013 [32], EN ISO 21528-2:2017 [33], and EN ISO 16649-2:2001 [34], respectively, as outlined in Commission Regulation (EC) No. 2073/2005 [35] on microbiological criteria for foodstuffs. For the analysis, 25 g of meat was aseptically transferred into a stomacher bag (Interscience, Saint Nom la Bretêche, France) containing 225 mL of sterile Maximum Recovery Diluent (MRD, CM0733, Oxoid, Basingstoke, UK) and homogenized using a stomacher (Lab Blender, Interscience, Shah Alam, Malaysia) for 2 min. Serial decimal dilutions were prepared in MRD solution, and 0.1 mL aliquots of each dilution were surface-inoculated onto the appropriate culture media. The TMVCs were enumerated on Plate Count Agar (PCA, Oxoid), whereas for the enumeration of Enterobacterales and *E. coli* counts, Violet Red Bile Glucose agar (VRBG, Oxoid) and Tryptone Bile X-glucuronide agar (TBX, Oxoid) were used, respectively. Incubation for TMVCs and Enterobacterales was performed at 37 °C for 48 h or 24 h, respectively, whereas incubation for *E. coli* was performed aerobically at 44 °C for 24 h. Following incubation, colonies with characteristic morphology were counted, and microbial populations were calculated as colony-forming units per gram (CFU/g).

### 2.9. Statistical Analysis

All statistical analyses were performed using R Statistical Software (v4.3.2) [36]. Descriptive statistics were calculated using the “psych” and “dplyr” packages [37,38]. The Shapiro–Wilk test was used to assess the normality of data distribution. In order to evaluate differences in beef quality traits between the 1st and 15th days of storage, a paired sample t-test was applied for normally distributed data (color parameters), while the Wilcoxon signed-rank test was used for non-normally distributed data (pH and texture parameters). The level of statistical significance was set at 0.05.

## 3. Results

### 3.1. Economic Performance of Studied Farms

Economic parameters used for analysis are included in Supplement File S2_Table S1. The average farm income, variable costs, and gross margin were EUR 152,873 ± 181,030, EUR 127,683 ± 136,959, and EUR 25,190 ± 44,373, respectively. On average, the gross margin per cow was EUR 49.3 ± 92.81; however, FB reported a negative gross margin per cow of EUR −26.63. Income for all farms was primarily derived from meat sales (74.1%) and subsidies (25.9%).

Feeding (60.6%) represented the largest portion of total variable costs, followed by labor (18.5%), land rent (12.6%), fuel (11.1%), veterinary costs (4.2%), utility bills (2.8%), and machinery costs (2.6%). The highest feeding costs were associated with males, averaging EUR 439.3 ± 142.78/year per animal. The feeding costs of each female were limited to EUR 194.0 ± 107.46/year.

Notably, the average gross margin, excluding subsidies, was negative at EUR −13,683 ± 4234. Similarly, the average gross margin per cow was also negative at EUR −130.5 ± 92.60.

### 3.2. Descriptive Statistics of Beef Quality Traits

Meat quality traits used for analyses are included in Supplement File S3_Dataset S2. Descriptive statistics for beef quality traits measured on the 1st and 15th days of storage are presented in Table 1. Mean pH values of beef on the 1st and 15th days of storage were 5.5 (±0.36) and 5.6 (±0.30), respectively. During this period, mean L* and a* values increased by 3.1% and 0.5%, respectively, while b* values decreased by 10.7%. Additionally, mean hardness 1 and hardness 2 values decreased by 13.9% and 19.5%, respectively.

The meat chemical composition and fatty acid profile are presented in Table 2. Beef had a mean protein content of 22.8 (±0.88)% and a fat content of 1.1 (±1.12)%, with 52.9 (±4.77)% being saturated fatty acids (SFAs), 44.6 (±4.71)% monounsaturated fatty acids (MUFAs), and 2.7 (±0.72)% polyunsaturated fatty acids (PUFAs). The average concentrations of n-3 and n-6 fatty acids were 0.4 (±0.10)% and 2.1 (±0.77)%, respectively.

The mean TMVCs and total Enterobacterales and *E. coli* counts were 5.0 (±0.57), 2.34 (±1.28), and 0.41 (±0.93) log10 CFU/g, respectively. Heavy metal concentrations (mg/kg) in bovine livers were below the maximum limits according to the European Commission: cadmium (Cd, 0.1 ± 0.11), lead (Pb, 0.05 ± 0.02), mercury (Hg, 0.2 ± 0.15), and arsenic (As, 0.02 ± 0.01).

### 3.3. Comparative Analysis of Beef Quality Traits Between the 1st and 15th Days of Storage

The results of the paired samples t-test and Wilcoxon signed-rank test, comparing beef quality traits between the 1st and 15th days of storage, are presented in Table 3 and Table 4, respectively. On day 15, beef samples exhibited significantly lower b* values (*p* < 0.01), hue angle (*p* < 0.001), cohesiveness (*p* < 0.01), and springiness (*p* < 0.01) compared to day 1. In contrast, the L* value was significantly higher (*p* < 0.01) on the 15th day of storage.

## 4. Discussion

This study aimed to evaluate the economic performance and to characterize meat quality traits of beef carcasses produced under extensive systems. We further assessed potential differences in meat color and texture parameters during the ageing process. Such research is highly relevant in Greece, given the economic challenges faced by the sector and the scarcity of data on meat quality.

In extensive systems, farm profitability could be affected by several factors, including herd size, breed, pasture quality and availability, weather and climate conditions, feeding costs, and beef prices [39]. Our findings indicate that farms in the Axios River Delta area had low profitability and depended significantly on subsidies to remain economically viable. Specifically, the economic analysis showed that FA was more profitable than the other farms and had a higher gross margin (EUR 130.93/cow), suggesting a better economic perspective for large farms; however, this finding needs to be supported by other studies that involve larger sample sizes. On the other hand, FB was not economically viable, as it exhibited a negative gross margin. This could be attributed to higher total variable costs per cow (81.2% and 130.6% compared to FA and FC, respectively). Additionally, feeding costs in FB—the greatest expenses category—were also higher by 101.6% and 134.9% compared to FA and FC, respectively. Our results are similar to those of other studies that highlight the low profitability of these systems and emphasize the necessity of European Union subsidies in ensuring a sustainable future for the beef sector [23,24,40,41]. Specifically, in two studies conducted in Greece, the profit without considering subsidies was negative at EUR −154.750/cow [23] and EUR −302/cow [24], respectively. Moreover, in the study of Michaličková et al. [41], the loss was even higher, as the profit, including subsidies, was negative at EUR −577/cow. Variations in results across studies could be attributed to differences in beef prices, feeding costs, and subsidy policies among countries. However, to achieve economic sustainability, it is essential to reduce dependency on external resources by implementing more efficient feeding practices that lower costs, increase animal productivity, and secure higher beef prices in the meat industry.

Nowadays, consumers focus on meat quality, with several factors influencing their purchasing decisions. Among these, beef color significantly affects their choices, as it plays a crucial role in consumer perception of quality. Generally, beef with a vibrant red color is perceived as higher quality compared to pale, discolored, or dark meat, which is often associated with spoilage or inferior quality [42]. Various factors influence meat color, such as genotype, diet, rearing system, pre- and post-slaughter handling conditions, and biochemical changes. Evaluating colorimetric parameters provides valuable insights into the quality of the final product [43]. Meat lightness (L*) is inversely related to its heme iron content, which increases with the animal’s age [44]. Variations in redness (a*) have been associated with metmyoglobin formation in meat, with low redness values indicating an undesirable dark coloration (green-brown) [45]. Additionally, meat fat color is evaluated based on the intensity of yellowness (b*), with higher values indicating a more pronounced yellow hue. In our study, the results of meat color assessment align with those of other studies on cattle raised under similar farming practices and fall within the preferred range (a* > 14.5, L* > 31.4, b* > 6.3) according to consumer preferences [11,46,47]. It is noteworthy that another study from Greece [25] reported lower a* values (12.88) and higher b* values (11.54) in beef from Katerini young bulls raised on pasture, indicating a less red and more brownish-yellowish appearance compared to the beef in the present study. This outcome may be explained by the different breeds included in the studies.

In our study, meat color changed during storage; b* value and hue angle decreased, whereas the L* value increased, indicating that beef maintained a more intense red color, which is associated with freshness, higher consumer appeal, and a brighter appearance. In contrast with our results, Humada et al. [48] observed an increase in b* value and hue angle, along with a decrease in L* value, as ageing time progressed. This difference may be attributed to higher average pH values (5.7) reported in their study, which may have led to a greater water-binding capacity, resulting in decreased light scattering and a lower L* value [48]. On the other hand, Ruiz de Huidobro et al. [49] reported no significant differences in meat color during storage. Overall, the enhanced brightness observed in the present study is particularly important, as meat produced by cattle reared in extensive systems often tends to be darker and less appealing to consumers [50].

Beef tenderness—especially its hardness—is among consumer preferences’ most important quality indicators. Studies suggest consumers are willing to pay higher prices for tender beef [47]. While the most commonly used method for assessing beef tenderness is the Warner–Bratzler Shear Force (WBSF), research suggests that Texture Profile Analysis (TPA) is more effective for predicting hardness in meat [51]. Our study used TPA on raw beef, making it challenging to compare our findings to most studies that use the WBSF method. Nevertheless, our results are consistent with Abd Rashid et al. [52], who reported similar tenderness values of 803 g in raw beef. In contrast, the study of Chinzorig and Hwang [53] reported higher levels of hardness (2000 gf) in the raw meat of *Longissimus thoracis* (LT) muscle after ageing for three days. Similarly, de Huidobro et al. [51] found higher hardness values (1860 gf) in 96 samples of *Longissimus dorsi* muscle six days postmortem. These differences may be attributed to variations in methodology and protocol design across different studies. Furthermore, meat springiness and cohesiveness significantly decreased during storage, which may be due to protein solubilization and the overall effects of ageing on meat texture [54]. Although no significant effects on hardness were observed over time, a slight decrease was noted, suggesting that meat softened during ageing [28,54].

Another important indicator of meat quality is pH, which is primarily influenced by the glycogen concentration in the animal’s muscles before slaughter. Specifically, low glycogen levels in muscles decrease lactic acid production, resulting in a higher final pH of meat [55]. The optimal pH range of beef is 5.3–5.7, while higher values have been associated with several undesirable characteristics in meat, including a darker color, coarse texture, increased water capacity, and reduced shelf life—all of which negatively impact its quality [29,56,57]. In our study, the average pH values were found within this optimal range, indicating effective pre-slaughter handling practices and suggesting that the final product aligns with consumer expectations and demands. Our results are similar to those reported for grass-fed [25,50,58] and grain-fed beef cattle in Greece [29].

Beef chemical composition and FA profile determine its nutritional value and are of significant interest to health professionals and consumers. Both traits are influenced by several factors, including breed, sex, muscle type, diet, and age [59,60,61]. Our findings are consistent with previous studies reporting that meat from extensively reared cattle has low fat content, ranging from 1.71% to 2.8% [25,59]. According to the FDA, this beef is classified as lean, containing less than 10 g of total fat per 100 g of meat [62]. This classification aligns with consumer trends, as more individuals are choosing healthier dietary options [63]. In contrast, meat protein content remains consistent regardless of the farming system, and our results align with previous research findings reporting protein levels ranging from 21.3% to 23.1% [64,65]. Furthermore, although several studies suggest that grass-fed cattle produce meat with high concentrations of PUFAs and n-3 fatty acids [60], this outcome was not always consistent [59]. In our study, PUFAs and linolenic acid, the primary n-3 fatty acid found in beef, were lower than those reported in other studies, which have documented PUFA levels ranging from 3.5% to 9.7% and linolenic acid levels between 0.91% and 1.67% [13,66,67]. This could be explained by the Limousin breed’s genetic tendency to store higher concentrations of SFA compared to other breeds [68], as well as variations in feeding practices among different studies [69].

Heavy metals and metalloids, such as Cd, Pb, Hg, and As, can adversely affect human health. Humans are primarily exposed to these metals through air, water, and food [70]. The most common toxic effects of heavy metal exposure include kidney, liver, and gastrointestinal disorders, nervous system damage, vascular injuries, skin lesions, immune system deficiencies, cancer, and congenital disabilities [71]. Among food commodities, the liver is one of the primary target organs for heavy metal accumulation [72]. Compared to our results, Yabe et al. [73] reported similar Pb levels (0.047 mg/kg) but 100–1000 times lower levels of Cd, Hg, and As in liver samples from extensively reared cattle in Zambia. Similarly, López-Alonso et al. [74] found that the concentrations of Cd, Pb, Hg, and As in extensively reared cattle in Spain were 10 to 100 times lower than those reported in our study. These differences could be associated with variations in grassland morphology among studies. Still, in the present study, none of the liver samples exceeded the maximum allowable levels for heavy metals in food products, as established by the Commission Regulation 2023/915 (0.2 mg Pb/kg wet weight in bovine offal; 0.5 mg Cd/kg wet weight in bovine liver) [75], indicating that the analyzed products are safe for consumption.

The enumeration of microbial indicators in meat products provides a simple, reliable, and rapid assessment of contamination from the environment and the overall hygiene conditions under which the food was produced, processed, and stored. TMVC is considered one of the most important process hygiene criteria for foodstuffs, including meat and meat products, as high counts indicate inadequate hygiene during processing [35]. TMVC values of ≥7 log_10_ CFU/g are considered the threshold for macroscopically detectable deterioration in meat quality [76]. In this study, TMVC levels were 2 log_10_ CFU/g below the 7 log_10_ CFU/g threshold, ensuring the microbiological stability of the meat when stored under refrigeration [77]. Regarding total Enterobacterales and *E. coli*, elevated levels in fresh meat are a consequence of improper handling during skinning and evisceration at slaughter, leading to contamination [78]. Additionally, Enterobacterales and *E. coli* can persist in the food processing environment and equipment, further contaminating final products [79]. From a human health perspective, these microbial indicators are essential for assessing the safety and hygienic status of meat, as their presence may reflect potential exposure to pathogenic microorganisms. *E. coli* is the most commonly used microbial indicator for monitoring fecal contamination in food and may signal the potential presence of enteric pathogens such as *Salmonella* or Shiga toxin-producing *E. coli* (STEC) strains [80]. Similarly, total Enterobacterales serves as an indicator of process integrity, reflecting the potential introduction of pathogens from the environment into food and signaling compromised hygiene practices or inadequate sanitation procedures [81]. The levels of total Enterobacterales and *E. coli* observed in this study indicate low levels of fecal contamination in fresh meat. Similar findings regarding the populations of TMVC, total Enterobacterales, and *E. coli* in beef cuts have also been reported in other studies [82,83]. For instance, Kukhtyn et al. [82] reported TMVC values of 4.88 log_10_ CFU/cm^2^ in refrigerated beef from Ukraine, while Kim et al. [83] found that 98.75% of beef samples from meat packing centers and 94.72% from retail meat shops in South Korea had TMVC values ≤ 1.0 × 10^6^ CFU/g. In the same study [83], *E. coli* counts in raw beef samples were approximately 1.0 × 10^1^ CFU/g, with values ranging from below detection to 8.0 × 10^1^ CFU/g.

While the significance of our results for Greek meat science is evident, this study also presents some limitations. Specifically, although this study focused on beef quality traits, a parallel analysis of pasture composition could help clarify the contribution of pasture characteristics to meat quality. Moreover, the economic analysis was based on data from only three farms within a specific region of Greece, which may limit the broader applicability of the findings. Additionally, incorporating more animal-related parameters in economic analysis, such as average daily gain and BW at slaughter, could provide a more comprehensive understanding of the factors influencing profitability and overall farm performance. Even though the beef samples assessed were representative of the studied area, future research involving larger sample sizes from more farms across different regions of Greece could improve the representativeness of the findings and further highlight the meat quality potential of extensive farming systems nationwide. In addition, such studies are of great interest not only for academic purposes but also for public engagement. Disseminating results through social media platforms can reach a broader audience beyond academia, including industry stakeholders, consumers, and the public [84]. In this context, a platform has been developed to share information about the current study with the public [85]. Still, continued efforts in science communication remain an important future direction. Enhancing transparency in this way could improve consumer perceptions, support local producers, and promote more sustainable agricultural practices.

## 5. Conclusions

The results of this study reveal that all participating farms located in the Axios River Delta face economic losses and rely on EU agricultural subsidies for their financial sustainability. Despite these financial challenges, microbiological, physical, and chemical analyses revealed that beef from extensive cattle farming systems in the protected area of the Axios River Delta possessed improved color parameters during storage, along with other favorable quality traits. Specifically, the meat was tender and had a low fat content, making it a nutritious option that aligns with consumer preferences for high quality. Furthermore, beef pH and microbial counts were within acceptable reference limits. Additionally, bovine livers showed heavy metal levels that were below the maximum limits set by the European Commission, confirming their safety for consumption. These findings suggest that beef from this region, produced under extensive systems within a European Natura 2000 protected area, offers a product with strong potential for value-added marketing based on its origin, sustainability, and biodiversity conservation. Overall, given the scarcity of research in this topic, this study makes a significant contribution to Greek meat science by providing valuable data on the economic viability and the quality of meat produced by extensive farming systems.

## Figures and Tables

**Table 1:** Descriptive statistics of beef quality traits, including pH, color (lightness—L*, redness—a*, yellowness—b*, chroma, hue angle), and texture (hardness 1, hardness 2, springiness, cohesiveness, chewiness) parameters measured on the 1st and 15th days of storage.

Trait	Day of Analysis	N	Mean (±SD ^1^)	Min ^2^	Max ^3^
pH	1st	36	5.5 (0.36)	4.63	6.03
	15th	35	5.6 (0.30)	4.78	6.10
Lightness—L*	1st	36	35.5 (1.78)	29.51	40.97
	15th	36	36.6 (1.41)	31.36	42.21
Redness—a*	1st	36	17.9 (2.11)	11.78	23.13
	15th	35	18.0 (2.24)	13.78	21.55
Yellowness—b*	1st	36	9.3 (2.32)	6.18	13.42
	15th	35	8.4 (1.78)	5.17	11.03
Chroma	1st	36	20.3 (2.77)	13.88	26.74
	15th	35	19.8 (2.08)	14.72	24.03
Hue angle	1st	36	0.5 (0.05)	0.37	0.57
	15th	35	0.4 (0.04)	0.36	0.52
Hardness 1 (g)	1st	33	1170.8 (849.39)	149.25	3114.81
	15th	33	905.5 (323.06)	415.77	1625.34
Hardness 2 (g)	1st	33	1027.6 (683.86)	138.59	2402.50
	15th	33	757.7 (278.89)	353.6	1412.85
Springiness	1st	33	0.8 (0.12)	0.51	1.03
	15th	33	0.7 (0.11)	0.52	0.93
Cohesiveness	1st	33	0.6 (0.10)	0.42	0.80
	15th	33	0.5 (0.09)	0.40	0.83
Chewiness (g)	1st	33	585.3 (469.34)	93.61	2114.8
	15th	33	353.4 (145.96)	124.64	624.94

^1^ SD = standard deviation; ^2^ Min = minimum; ^3^ Max = maximum.

**Table 2:** Descriptive statistics of meat chemical composition (moisture, protein, fat, collagen, salt, ash) and fatty acid profile (SFAs, UFAs, MUFAs, PUFAs, n-3 and n-6 fatty acids) assessed on the 15th day of storage.

Trait	N	Mean (±SD ^1^)	Min ^2^	Max ^3^
Moisture (%)	33	75.8 (1.86)	72.10	79.0
Protein (%)	33	22.8 (0.88)	20.20	24.40
Fat (%)	33	1.1 (1.12)	0.01	5.70
Collagen (%)	33	1.5 (0.46)	0.10	2.30
Salt (%)	33	0.8 (0.80)	0.01	3.60
Ash (%)	33	1.1 (0.74)	0.30	3.0
SFAs ^4^ (%)	32	52.9 (4.77)	42.12	62.10
UFAs ^5^ (%)	32	47.3 (4.91)	37.90	57.88
MUFAs ^6^ (%)	32	44.6 (4.71)	35.72	55.09
PUFAs ^7^ (%)	32	2.7 (0.72)	1.18	4.68
Myristic acid (C14:0, %)	32	2.2 (0.37)	1.54	2.89
Pentadecanoic acid (C15:0, %)	26	0.5 (0.14)	0.25	0.77
Palmitoleic acid (C16:1, %)	32	2.8 (0.77)	1.79	5.89
Heptadecanoic acid (C17:0, %)	32	0.9 (0.16)	0.60	1.24
Cis-10 Heptadecenoic acid (C17:1, %)	19	0.4 (0.13)	0.25	0.67
Stearic acid (C18:0, %)	32	24.9 (3.93)	14.67	32.86
Palmitic acid (C16:0, %)	32	23.8 (1.65)	19.94	26.79
Elaidic acid (C18:1 n-9 trans, %)	30	1.9 (0.52)	0.76	3.10
Oleic acid (C18:1 n-9 cis, %)	32	38.9 (4.17)	30.84	46.91
Linoleic acid (C18:2 n-6 cis, %)	32	0.2 (0.12)	0.01	0.44
Arachidic acid (C20:0, %)	20	0.03 (0.01)	0.01	0.04
Linolenic acid (C18:3 n-3, %)	20	0.4 (0.10)	0.19	0.53

^1^ SD = standard deviation, ^2^ Min = minimum, ^3^ Max = maximum, ^4^ SFAs = saturated fatty acids, ^5^ UFAs = unsaturated fatty acids, ^6^ MUFAs = monounsaturated fatty acids, ^7^ PUFAs = polyunsaturated fatty acids.

**Table 3:** Paired samples t-test results for color parameters (lightness—L*, yellowness—b*, hue angle) of beef samples between the 1st and the 15th days of storage.

Trait	Day of Storage	Mean (±SD ^1^)	t ^2^	df ^3^	*p*-Value
Lightness-L*	1st	35.50 (1.78)	2.834	34	0.007
	15th	36.59 (1.41)			
Yellowness-b*	1st	9.28 (2.32)	−2.862	34	0.008
	15th	8.37 (1.78)			
Hue Angle	1st	0.48 (0.05)	4.246	34	<0.001
	15th	0.43 (0.04)			

^1^ SD = standard deviation, ^2^ t = t-statistic, ^3^ df = degrees of freedom.

**Table 4:** Wilcoxon signed-rank test results for texture parameters (cohesiveness, springiness) of beef samples between the 1st and 15th days of storage.

Trait	Day of Storage	Median (IQR ^1^)	*p*-Value
Cohesiveness	1st	0.63 (0.13)	0.008
	15th	0.52 (0.10)	
Springiness	1st	0.81 (0.14)	0.006
	15th	0.70 (0.17)	

^1^ IQR = interquartile range.

## Data Availability

The dataset is available upon request from the authors.

## Supplementary Materials

The following supporting information can be downloaded at: https://www.mdpi.com/article/10.3390/ani15111601/s1, Supplement File S1_Dataset S1: Questionnaire with technical and economic data; Supplement File S2_Table S1: Total dataset of economic parameters used for analyses; Supplement File S3_Dataset S2: Total dataset of meat traits used for analyses.
