# Supplementary material for: Economic Performance and Meat Quality Traits of Extensively Reared Beef Cattle in Greece
Source: Animals (Basel). 2025 May 29;15(11):1601. doi: 10.3390/ani15111601 (PMC12153860; doi:10.3390/ani15111601)
Supplement: Supplementary file 1 [file animals-15-01601-s001.zip › Supplement File S1_Dataset S1.pdf]

QUESTIONNAIRE

GENERAL INFORMATION

- Date of Visit: ____
- Owner's Full Name: ____
- Age: ____
- Phone/Email: ____
- Location: ____
- Farm Code: ____
- Farming System: ____
- Main Activity: ____

PERSONELL

- Number of Employees: ____
- Responsibilities: ____
- Part-Time Employment: ____
- Full-Time Employment: ____

FARM AND ANIMALS

Breed and origin

- Breeding females: ______________________________________
- Sires: ______________________________________
- Fattening calves: ______________________________________

Farm

- Breeding and Fattening: ____
- Fattening of calves (local breed livestock): ______________________________
- Fattening of calves (imported livestock): ______________________________

- Fattening of Holstein calves: ____
- Number of breeding females: ____
- Replacement females: ____
- Number of sires: ____
- Number of fattening calves: ____
- Total number of livestock: ____

Which records are available on the farm?

- Body Weights (BW): ____
- Feed Analyses: ____
- Income-Expenses: ____
- Other: ____

REPRODUCTIVE MANAGEMENT

- Heifers - Age at mating: ____

Use of Artificial Insemination: ____

- Bull Semen (breed)____
- Synchronization Protocols: ____

Natural Mating:

- Bull breed: ____

Calving

- Calving Interval: ____
- Breeding period: ____
- Calving period: ____
- Pregnancy diagnosis – method: _______________________________________
- Estrus detection – method: ____

STABLE

Description of fattening facilities

- Size (m^2): ____
- Floor & bedding type: ____

- Ventilation type: ____
- Number of boxes: ____
- Number of animals/box: ____
- Feeding – providing method: ______________________________________
- Frequency of manure removal - Method: ____________________________

Description of adults' facilities

- Size (m^2): ____
- Floor & bedding type: ____
- Ventilation type: ____
- Number of boxes: ____
- Number of animals/box: ____
- Feeding – providing method: ______________________________________

GRAZING AND FEEDING

- Grazing period: ____
- Grazing area (acres): ____
- Groups of animals that graze: _____________________________________
- Duration (hours/day): ____
- Walking distance (km/day): _______________________________________

Adults feeding

**Roughages**	**Kg/ animal**		**€/kg**
Corn silage			
Alfalfa hay			
Straw			
Other			
**Concentrates**	**%**	**€/kg**	**Total (Kg)/ animal**
Corn			
Barley			
Wheat			
Wheat bran			
Soybean meal			
Sunflower meal			
Cottonseed cake			
Fat			
Vitamin-mineral supplement			
Other			

Replacement heifers -feeding

**Roughages**	**Kg/ animal**		**€/kg**
Corn silage			
Alfalfa hay			
Straw			
Other			
**Concentrates**	**%**	**€/kg**	**Total (Kg)/ animal**
Corn			
Barley			
Wheat			
Wheat bran			
Soybean meal			
Sunflower meal			
Cottonseed cake			
Fat			
Vitamin-mineral supplement			
Other			

Feeding - calves until weaning

- Duration of lactation: ____
- Use of milk replacer: ____
- Age of starting roughage feeding: ____________________________________
- Age of starting concentrate feeding: __________________________________
- BW at weaning: ____

**Roughages**	**Kg/ animal**		**€/kg**
Corn silage			
Alfalfa hay			
Straw			
Other			
**Concentrates**	**%**	**€/kg**	**Total (Kg)/ animal**
Corn			
Barley			
Wheat			
Wheat bran			
Soybean meal			
Sunflower meal			
Cottonseed cake			
Fat			
Vitamin-mineral supplement			
Other			

Feeding - calves from weaning until entering the final fattening stage

**Roughages**	**Kg/ animal**		**€/kg**
Corn silage			
Alfalfa hay			
Straw			
Other			
**Concentrates**	**%**	**€/kg**	**Total (Kg)/ animal**
Corn			
Barley			
Wheat			
Wheat bran			
Soybean meal			
Sunflower meal			
Cottonseed cake			
Fat			
Vitamin-mineral supplement			
Other			

Feeding - calves in final fattening stage

**Roughages**	**Kg/ animal**		**€/kg**
Corn silage			
Alfalfa hay			
Straw			
Other			
**Concentrates**	**%**	**€/kg**	**Total (Kg)/ animal**
Corn			
Barley			
Wheat			
Wheat bran			
Soybean meal			
Sunflower meal			
Cottonseed cake			
Fat			
Vitamin-mineral supplement			
Other			

Slaughtering Data

- Age and body weight of males entering the final fattening stage: ____

- Age and body weight of males at the end of the final fattening period: ____

- Age and body weight of females entering the final fattening stage: ____

- Age and body weight of females at the end of the final fattening period: ____

Month	Number of Slaughtering	Average BW at slaughter	Average Carcass Weight
Jan.			
Feb.			
Mar.			
Apr.			
May			
Jun.			
Jul.			
Aug.			
Sept.			
Oct.			
Nov.			
Dec.			

Beef price/kg:

Type of vaccinations performed:

- *Mannheimia*: ____
- BRSV: ____
- BVD: ____
- IBR: ____
- *Clostridium* spp. : ____
- Other: ____

Deworming:

- Calves: ____
- Males: ____
- Replacement heifers:____
- Adults: ____
- Substance: ____
- Frequency of administration/year: __________________________________

- Parasitological examinations: ____

HEALTH

Adults

- Abortions (number of animals/year): _________________________________
- Lameness (number of animals/year): _________________________________
- Pneumonia (number of animals/year): ________________________________
- Diarrhea (number of animals/year): __________________________________
- Death/removal (number of animals/year): _____________________________
- Other: ____

Calves < 1 month

- Pneumonia (number of animals/year): ________________________________
- Diarrhea (number of animals/year): __________________________________
- Death/removal (number of animals/year): _____________________________
- Other: ____

Calves 1 month-6 months

- Pneumonia (number of animals/year): ________________________________
- Diarrhea (number of animals/year): __________________________________
- Death/removal (number of animals/year): _____________________________
- Other: ____

Calves 6 months to the stage of entering the final fattening stage

- Pneumonia (number of animals/year): ________________________________
- Diarrhea (number of animals/year): __________________________________
- Death/removal (number of animals/year): _____________________________
- Other: ____

Calves in final fattening stage

- Pneumonia (number of animals/year): ________________________________
- Diarrhea (number of animals/year): __________________________________
- Death/removal (number of animals/year): _____________________________
- Other: ____

ECONOMIC DATA

- Production (tons of meat): ____
- Income from sales of live animals (€/year): _____________________________
- Income from sales of slaughtered animals (meat) (€/year): ________________

Income from subsidies

- Subsidy last year (€): ____
- Subsidy per animal (€): ____

Costs for animal purchases (€/year): _____________________________________

Costs for employed personnel

- Workers' salaries (€/year): ____
- Seasonal personnel (€/year): ____

Costs - Land rent

- Grazing land (€/acre): ____
- Cultivated land (€/total): ____

Fuel (€/year): ____

Utility bills

- Electricity (€/year): ____
- Water (€/year): ____
- Telephone (€/year): ____
- Other (€/year): ____

Veterinary costs

- Vaccinations (€/animal): ____
- Deworming (€/animal): ____
- Reproduction (€/animal): ____
- Other (€/animal): ____

Costs - grazing land

- Seeds (€/year): ____
- Fertilizer (€/year): ____
- Other (€/year): ____

Expenses for maintenance of facilities and machinery (€/year): _________________

Expenses for the purchase of new equipment (€/year): _________________________
