# Supplementary material for: Economic Performance and Meat Quality Traits of Extensively Reared Beef Cattle in Greece
Source: Animals (Basel). 2025 May 29;15(11):1601. doi: 10.3390/ani15111601 (PMC12153860; doi:10.3390/ani15111601)
Supplement: Supplementary file 1 [file animals-15-01601-s001.zip › Supplemnet File S2_Table S1.pdf]

Table S1. Economic parameters of studied farms.

Trait	Farm A	Farm B	Farm C
Gross margin (EUR)	76,390	- 2,130	1,311
Gross margin per cow (EUR)	152.78	-26.63	21.85
Income (EUR)	361,500	59,890	37,230
Income from meat sales (%)	74.69	75.14	72.52
Income from subsidies (%)	25.3	24.9	27.5
Variable costs (EUR)	285,110	62,000	35,919
Feeding costs (%)	52.7	65.2	63.7
Labour costs (%)	18.5	-	-
Renting land costs (%)	10.5	15.5	11.7
Fuel costs (%)	7.0	12.9	13.4
Veterinary costs (%)	10.5	1.6	0.6
Utility bills costs (%)	0.4	1.6	6.4
Machinery costs (%)	0.4	3.2	4.2
